# Activation of the DNA Damage Response Is a Conserved Function of HIV-1 and HIV-2 Vpr That Is Independent of SLX4 Recruitment

**DOI:** 10.1128/mBio.01433-16

**Published:** 2016-09-13

**Authors:** Oliver I. Fregoso, Michael Emerman

**Affiliations:** Division of Human Biology, Fred Hutchinson Cancer Research Center, Seattle, Washington, USA

## Abstract

There has been extraordinary progress in understanding the roles of lentiviral accessory proteins in antagonizing host antiviral defense proteins. However, the precise primary function of the accessory gene Vpr remains elusive. Here we suggest that engagement with the DNA damage response is an important function of primate lentiviral Vpr proteins because of its conserved function among diverse lentiviral lineages. In contrast, we show that, for HIV-1, HIV-2, and related Vpr isolates and orthologs, there is a lack of correlation between DNA damage response activation and interaction with the host SLX4 protein complex of structure specific endonucleases; some Vpr proteins are able to interact with SLX4, but the majority are not. Using the clustered regularly interspaced short palindromic repeat (CRISPR)/Cas9 method to knock out SLX4, we formally showed that HIV-1 and HIV-2 Vpr orthologs can still activate the DNA damage response and cell cycle arrest in the absence of SLX4. Together, our data suggest that activation of the DNA damage response, but not SLX4 interaction, is conserved and therefore indicative of an important function of Vpr. Our data also indicate that Vpr activates the DNA damage response through an SLX4-independent mechanism that remains uncharacterized.

## IMPORTANCE

HIV-1 and HIV-2 belong to a family of viruses called lentiviruses that infect at least 40 primate species, including humans. Lentiviruses have been circulating in primates for at least 5 million years. In order to better fight HIV, we must understand the viral and host factors necessary for infection, adaptation, and transmission of these viruses. Using the natural variation of HIV-1, HIV-2, and related lentiviruses, we have investigated the role of the DNA damage response in the viral life cycle. We have found that the ability of lentiviruses to activate the DNA damage response is largely conserved. However, we also found that the SLX4 host factor is not required for this activation, as was previously proposed. This indicates that the DNA damage response is an important player in the viral life cycle, and yet the mechanism(s) by which HIV-1, HIV-2, and other primate lentiviruses engage the DNA damage response is still unknown.

## INTRODUCTION

HIV-1 and other primate lentiviruses encode accessory proteins that enhance viral infectivity (1). These include Vpr, Vif, Vpu, and Nef. In addition, a subset of lentiviruses, including HIV-2, also encode a paralog of Vpr called Vpx. In general, accessory proteins recruit host proteins that are important for viral replication and/or antagonize antiviral factors. Vpr is an accessory protein that is found in all extant primate lentiviruses (2, 3). Vpr has also been shown to be important for HIV and simian immunodeficiency virus (SIV) pathogenesis *in vivo* (4–7). However, despite its persistence throughout primate lentiviral evolution and its importance in the viral life cycle, the precise primary function of Vpr remains unclear.

Many roles have been ascribed to Vpr, including long terminal repeat (LTR) transactivation, preintegration complex nuclear import, cellular apoptosis, and G_2_ cell cycle arrest (8). In addition, HIV-1 Vpr activates DNA damage response pathways and interacts with host complexes involved in the sensing and repair of damaged DNA. HIV-1 Vpr activates ATR (ataxia-telangiectasia and Rad3-related) protein and its downstream target Chk1 (9–11), a major signaling pathway of the DNA damage response. This leads to activation of DNA damage response markers, such as the accumulation of replication protein A (RPA), γH2AX (12, 13), and FANCD2 (14), at chromatin-associated nuclear foci, as well as to G_2_ cell cycle arrest. The interaction of Vpr with the host Cul4^DCAF1^ ubiquitin ligase complex is required for many of these Vpr-related phenotypes (15, 16).

Recently, HIV-1 Vpr was shown to interact with an additional DNA damage response complex: the SLX4 complex of structure-specific endonucleases (14). This interaction was described as being responsible for the effects of Vpr on cell cycle arrest as well as for suppression of an antiviral interferon response otherwise elicited by HIV-1 (14). The SLX4 complex is comprised of 12 proteins that together resolve DNA lesions formed during DNA replication stress and/or homologous recombination repair (17–19). Vpr interacts with this complex through a direct interaction with the C terminus of the SLX4 scaffold protein (14). This interaction is thought to hyperactivate the endonuclease activity of the SLX4 complex through increased phosphorylation of the SLX4-complex-associated PLK1 kinase, resulting in nonspecific DNA cleavage and subsequent activation of ATR and cell cycle arrest to control or resolve this damage. Moreover, it was shown that Vpr proteins from lentiviruses infecting African green monkeys and related primates also interact with African green monkey SLX4, suggesting that this is a conserved function for Vpr (20).

Understanding the conservation of function (or lack thereof) within the family of primate lentiviruses can help to identify important functions of viral accessory proteins (21). However, one major caveat with respect to experiments using proteins from diverse primate lentiviruses to assay functions in human cells is that, due to the evolutionary arms race between the virus and its host, they may have evolved to be species-specific interactions (22). This can be circumvented by studying HIV-1 and HIV-2, which both naturally infect humans and yet have distinct evolutionary histories. HIV-1 arose from the cross-species transmission of SIV infecting chimpanzees (SIVcpz) into humans (23, 24). SIVcpz is a recombinant virus corresponding to SIVmus and SIVrcm, with Vpr originating from a virus related to SIVrcm (25). HIV-2 arose from a distinct lineage of primate lentiviruses, namely, those infecting sooty mangabey (SIVsmm) (26, 27). Thus, HIV-1 and HIV-2 present a powerful toolset to look more in depth at conservation of function between divergent lentiviruses, as they have both evolved to infect humans but have distinct parental viruses and evolutionary histories.

Here, we took advantage of the unique evolutionary history of HIV-1 and HIV-2 to investigate the requirements and importance of engagement of the DNA damage response by primate lentiviruses. We found that activation of the DNA damage response is a conserved outcome of expression of Vpr orthologs from both HIV-1 and HIV-2 and that it is largely conserved among primate lentiviral Vpr proteins. However, we found that, unlike activation of the DNA damage response, the interaction of Vpr and SLX4 is variable among HIV-1 and HIV-2 isolates, thus identifying a lack of correlation between Vpr-mediated DNA damage response activation and SLX4 binding. Furthermore, clustered regularly interspaced short palindromic repeat (CRISPR)/Cas9 knockout cells for SLX4 showed that the DNA damage response and cell cycle arrest induced by Vpr are not dependent on the interactions of Vpr and SLX4, in contrast with previous reports (14, 20). Thus, our results argue that the mechanism by which lentiviral Vpr proteins induce a DNA damage response has yet to be determined.

## RESULTS

### HIV-1 and HIV-2 Vpr proteins differentially engage the DNA damage response.

By testing distinct Vpr orthologs for their ability to interact with and activate the DNA damage response in the same natural host, we sought to determine if these functions are conserved and therefore important for primate lentiviruses. Thus, since HIV-1 and HIV-2 both replicate in their human host and yet have very different primate origins, we reasoned that assaying the ability of Vpr proteins from both viruses to activate the DNA damage response and/or to interact with SLX4 would help shed light on the importance of these functions for primate lentiviruses.

**FIG 1:**
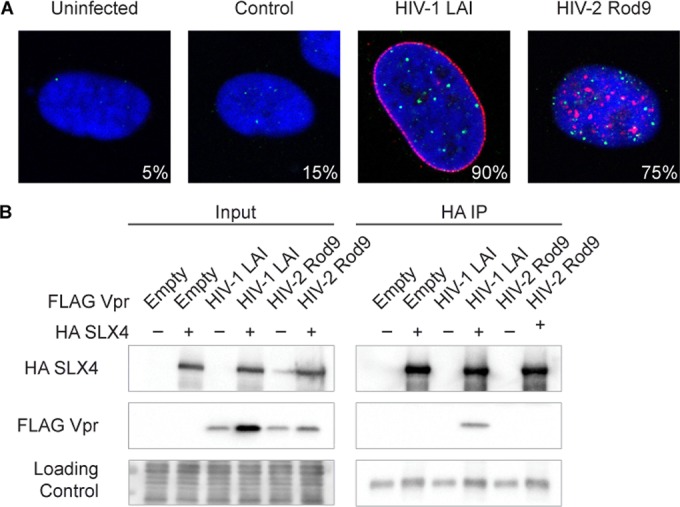
HIV-1 and HIV-2 Vpr differentially engage the host DNA damage response. (A) Representative immunofluorescence images of U2OS cells expressing 3× FLAG-tagged Vpr, control empty vector (no Vpr), or uninfected cells. FANCD2 foci show activation of the DNA damage response. Blue (DAPI) shows the nuclei, 3× FLAG Vpr is shown in red, and FANCD2 is shown in green. Values indicate percentages of FANCD2-positive cells (more than 6 FANCD2 foci) that were also Vpr positive. For negative controls, values are simply the percentages of FANCD2-positive cells in the entire field (*n* = >15). (B) HA-tagged human SLX4 and FLAG-tagged Vpr were transiently coexpressed in 293T cells (left panels), and immunoprecipitations against the HA tag were performed (right panels). The loading control was either whole-cell stain (Input) or IgG heavy chain (HA IP) (note that there is a small amount of sample spillover in the HIV-2 Rod9-negative SLX4 input lane from the adjacent lane). See Fig. S1 in the supplemental material for related data.

To test for activation of the DNA damage response by HIV-1 (LAI) and HIV-2 (Rod9) Vpr, we generated an adeno-associated virus (AAV) vector system expressing 3× FLAG-tagged Vpr, which allowed consistent and robust Vpr expression in culture cells. We assayed for activation of the DNA damage response by imaging cells for the formation of FANCD2 foci on chromatin (28) and also monitored the cells for cell cycle status. It has been previously shown that HIV-2, like HIV-1, causes G_2_ arrest in human cells (29, 30), a result that we were able to reproduce with our AAV expression system with no appreciable background activation (see Fig. S1 in the supplemental material). FANCD2 foci were visualized by indirect immunofluorescence, where the presence of discrete nuclear foci is indicative of DNA damage response activation. Consistent with the literature (14), overexpression of HIV-1 Vpr led to an increase in FANCD2 focus formation compared to results seen with the empty vector control (Fig. 1A). We further found here that HIV-2 Vpr also increases FANCD2 focus formation, suggesting that both of these divergent viral accessory proteins can activate the DNA damage response.

Previous studies had suggested that the interaction of Vpr with the SLX4 scaffold of endonucleases correlated with the cell cycle effects of Vpr (14, 20). As Vpr from both HIV-1 and HIV-2 causes FANCD2 focus formation and G_2_ arrest in human cells, we would expect that both orthologs would be able to recruit SLX4 if SLX4 recruitment were required for this activation. We therefore looked at the ability of HIV-1 and HIV-2 Vpr to interact with SLX4 by coimmunoprecipitation (co-IP). We transiently coexpressed hemagglutinin (HA)-tagged human SLX4 (N-terminally truncated for enhanced expression, as the interaction of HIV-1 Vpr with SLX4 has been mapped to the C terminus of SLX4) and FLAG-tagged Vpr orthologs in human 293T cells and performed an immunoprecipitation against HA-SLX4. As has been previously shown (14), HIV-1 Vpr (LAI strain) interacts with human SLX4 (Fig. 1B). However, we could not detect an interaction of HIV-2 Vpr with human SLX4 (Fig. 1B), suggesting that these human lentiviruses engage the DNA damage response independently of SLX4.

**FIG 2:**
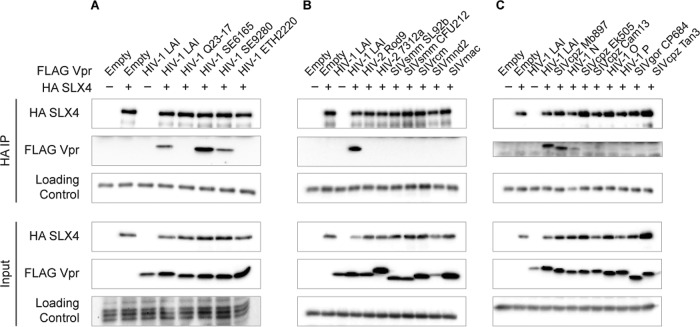
Interactions of HIV-1 Vpr with SLX4 are variable. Vpr from multiple isolates closely related to HVI-1 or HIV-2 were tested for their ability to interact with human SLX4 by coimmunoprecipitation. (A) HIV-1 group M Vpr isolates: HIV-1 LAI (subtype B), Q23-17 (subtype A), SE6165 (subtype G), SE9280 (subtype J), and ETH2220 (subtype C). (B) HIV-2 and related Vpr orthologs. (C) HIV-1 groups and ancestral SIVcpz and SIVgor Vpr orthologs. 3× FLAG Vpr was transiently coexpressed with HA-tagged SLX4 in 293T cells (lower panels), and immunoprecipitations against the HA tag were performed (upper panels). HIV-1 LAI Vpr was used as a positive control. The loading control was whole-cell stain (Input), tubulin (Input), or IgG heavy chain (HA IP). See Fig. S2 in the supplemental material for related data, including longer exposures for FLAG-Vpr following HA-IP.

In order to determine whether or not the interaction of HIV-1 Vpr with SLX4, or the lack of interaction of HIV-2 Vpr with SLX4, was specific to the HIV-1 LAI and HIV-2 Rod9 isolates tested, we assayed Vpr from additional HIV-1 and HIV-2 strains and related viruses for the ability to interact with SLX4 (see Fig. S2A and B in the supplemental material for a schematic representation of the phylogeny of Vpr orthologs used in this study). We first looked at other HIV-1 group M Vpr strains in addition to HIV-1 LAI (subtype B): strains Q23-17 (subtype A), SE6165 (subtype G, a primary isolate), SE9280 (subtype J, a primary isolate), and ETH2220 (subtype C, a primary isolate). We found that while three (LAI, SE6165, and SE9280) of the five HIV-1 Vpr isolates that we tested interacted with SLX4, two (Q23-17 and ETH2220) of them did not (Fig. 2A; see also Fig. S2C), indicating variability within closely related viral proteins with respect to their ability to interact with SLX4. Moreover, we assayed Vpr orthologs related to HIV-2 Rod9 (group B), including HIV-2 7312a (group A), SIVs infecting the sooty mangabey (SIVsmm SL92B and CFU212, which share an ancestor with HIV-2), and rhesus macaque (SIVmac239, which was derived directly from SIVsmm). Similarly to the results seen with HIV-2 Rod9, we found that none of the HIV-2 related Vpr proteins tested were able to recruit SLX4 (Fig. 2B; see also Fig. S2D). Thus, the ability of Vpr to interact with SLX4 could not be detected in any of the HIV-2 or related viruses tested and was variable in HIV-1 strains.

To cover the breadth of HIV-1 and HIV-2 sequence diversity and evolution, we looked at additional Vpr orthologs that diverged from the most recent common ancestors with HIV-1 group M and HIV-2. We looked at Vpr proteins from HIV-1 groups N, O, and P (using codon-optimized consensus sequences for groups N and O), as well as Vpr from SIV infecting chimpanzee (SIVcpz) and gorilla (SIVgor) (Fig. 2C; see also Fig. S2E in the supplemental material) that share common ancestors with all HIV-1 groups (M, N, O, and P). In addition, we looked at SIV Vpr from mandrill (SIVmnd2) and the red-capped mangabey (SIVrcm) (Fig. 2B), which phylogenetically clusters with HIV-2 Vpr (2). We found among these Vpr orthologs variability similar to what was found within HIV-1 and HIV-2 Vpr proteins—a few Vpr proteins (including Vpr from SIVcpz MB897 and HIV-1 group N) interacted with human SLX4, while the majority did not (Fig. 2). These results suggest that interaction of SLX4 with Vpr is variable and is not conserved by HIV-1 or related primate lentiviruses and therefore might not be necessary for the conserved functions of Vpr.

### CRISPR/Cas9 knockout of SLX4 indicates that recruitment of SLX4 is not required for activation of the DNA damage response by either HIV-1 or HIV-2 Vpr.

**FIG 3:**
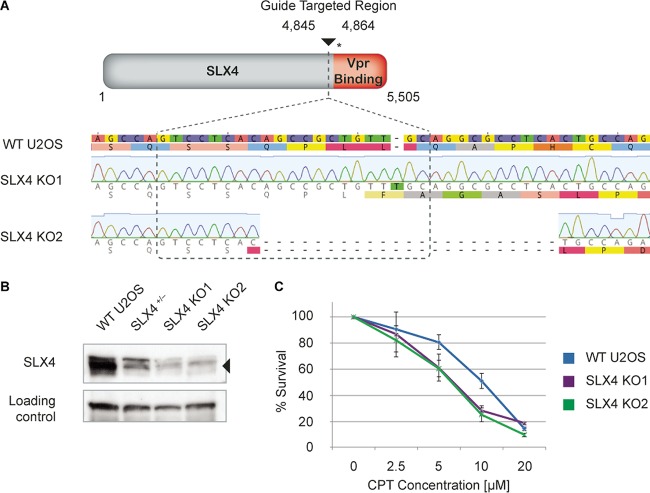
CRISPR/Cas9 SLX4 knockout cells are functionally deficient for SLX4. U2OS cells were targeted for SLX4 using CRISPR/Cas9-mediated genome editing. (A) Schematic representation of CRISPR/Cas9 targeting of SLX4. The SLX4 target region is represented with a solid inverted triangle, and the base pair positions spanning the guide RNA target are noted. The region of SLX4 where Vpr is proposed to bind is indicated in red. The asterisk (*) indicates a representative premature termination codon introduced by Cas9-mediated mutagenesis. For example, when a T is introduced, as indicated at the bottom of the panel, the introduction of the T results in 10 additional premature termination codons that are markers for nonsense-mediated mRNA decay. Results of sequencing of genomic DNA from a single-cell clone of wild-type U2OS cells (WT) and two single-cell clones of SLX4-targeted cells (SLX4 KO1 and SLX4 KO2) are shown. SLX4 KO1 has an inserted T present in all alleles, while SLX4 KO2 has a 22-bp deletion present in all alleles. (B) Western blot of U2OS whole-cell lysates for the wild type (WT), a heterozygous clone with both WT and mutant SLX4 alleles (SLX4^+/−^), and two single-cell clones that are mutated in all SLX4 alleles (SLX4 KO1 and SLX4 KO2). (C) Sensitivities of the WT, SLX4 KO1, and SLX4 KO2 strains to the interstrand cross-linking agent camptothecin (CPT) were tested by incubating cells for 7 days in the presence of CPT at the indicated concentrations. Survival was analyzed by crystal violet staining for live cells compared to no drug treatment. *n* = 3; standard deviations (SD) are shown. See Fig. S3 in the supplemental material for related data.

The results showing that both HIV-1 and HIV-2 activate the DNA damage response but that only some HIV-1 Vpr isolates interact with SLX4 indicate either that HIV-1 and HIV-2 Vpr orthologs activate the DNA damage response through distinct mechanisms, or that SLX4 recruitment is not required for activation of the DNA damage response by either HIV-1 or HIV-2 Vpr. To differentiate between these two hypotheses, we knocked out SLX4 using CRISPR/Cas9 in U2OS and 293T cells (Fig. 3 and Fig. S3 in the supplemental material, respectively) and asked if Vpr was still able to activate the DNA damage response in these cells (a schematic of the targeting strategy is shown in Fig. 3A).

Single-cell clones of U2OS and 293T cells were isolated, and the CRIPSR/Cas9-induced mutations were analyzed by sequencing the genomic locus to identify cell clones in which all alleles of SLX4 were disrupted. For several clones, we further characterized the mutations in individual alleles by TA cloning of these PCR products and sequencing at least 10 individual colonies from each clone. We identified two clones from each cell line that showed mutations in SLX4 in both alleles, with no detection of wild-type SLX4 sequences (Fig. 3A; see also Fig. S3A in the supplemental material). In each of these clones, the deletions introduced premature termination codons that would truncate the protein before the Vpr binding site and that should target SLX4 mRNA for nonsense-mediated mRNA decay (Fig. 3A). As a confirmation of the genomic mutations, these clones were analyzed by Western blotting for SLX4 protein expression using a polyclonal antibody (Fig. 3B). In all cases, full SLX4 gene disruption resulted in a lack of SLX4 protein expression. In addition, we identified a U2OS clone that had one mutant and one wild-type SLX4 allele which showed intermediate SLX4 expression by Western blotting (Fig. 3B); the results argue that the Western blot signal seen at approximately the size of SLX4 in the knockout clones was due to background from the antibody rather than retention of an allele of SLX4. As further confirmation of the genomic knockout, we functionally tested for SLX4 deficiency by assaying U2OS knockout clones for sensitivity to the interstrand cross-linking agent camptothesin (CPT), as SLX4-deficient cells are retarded in their ability to repair CPT-induced DNA lesions, leading to cell death (31, 32). Both U2OS clones showed an increased sensitivity to CPT (Fig. 3C), and similar results were found with mitomycin C (data not shown). Together, the results indicate that these cells are knocked out for SLX4.

**FIG 4:**
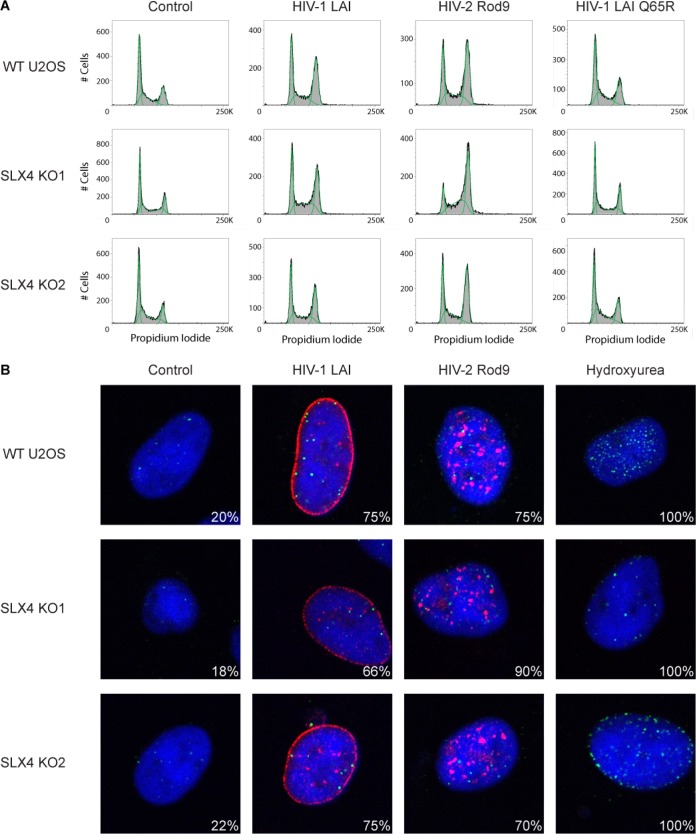
HIV-1 and HIV-2 Vpr can activate the DNA damage response in the absence of SLX4. SLX4 WT, KO1, and KO2 U2OS cells were infected with AAV vectors containing HIV-1 or HIV-2 Vpr and assayed for (A) cell cycle status and (B) FANCD2 focus formation. Virus without Vpr was used as a negative control (A and B), as was HIV-1 LAI Q65R Vpr (A). Note the increase in the G2 fraction of DNA (the second peak) in HIV-1 and HIV-2 Vpr transduction relative to the control and HIV-1 Vpr LAI Q65R transduction in all cells. (B) Hydroxyurea (250 µM, 24 h) was used as a positive control for FANCD2 foci. Blue (DAPI) shows the nuclei, 3× FLAG Vpr is shown in red, and FANCD2 is shown in green. Percentages were calculated as described for Fig. 1A (*n* = >7). See Fig. S3 in the supplemental material for related data.

We expressed HIV-1 and HIV-2 Vpr in SLX4-deficient cells and assayed for Vpr-mediated cell cycle arrest (in U2OS and 293T cells) and the formation of FANCD2 foci (in U2OS cells). We found that in all cases, the absence of SLX4 did not alter the ability of HIV-1 or HIV-2 Vpr to activate the DNA damage response, as Vpr proteins from both viruses were still able to induce G_2_ arrest (Fig. 4A; see also Fig. S3B in the supplemental material). Similarly, Vpr proteins from both viruses also caused FANCD2 focus formation in the SLX4 knockout cells (Fig. 4B). This demonstrates that activation of the DNA damage response by both HIV-1 and HIV-2 Vpr orthologs is independent of SLX4 recruitment.

**FIG 5:**
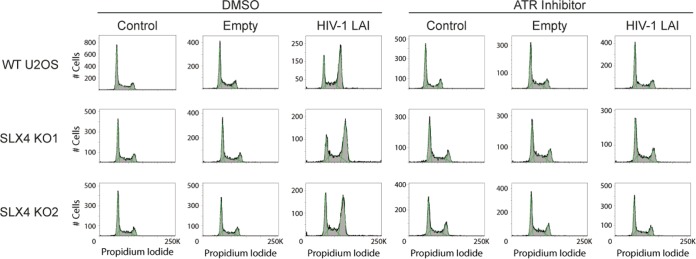
Vpr-mediated activation of the DNA damage response still proceeds through ATR in the absence of SLX4. SLX4 WT, KO1, and KO2 U2OS cells were infected with AAV vectors containing HIV-1 Vpr or controls and assayed for cell cycle status in the presence of 10 µM VE-821 (ATR inhibitor) for 20 h or dimethyl sulfoxide (DMSO) control.

While we have found that Vpr-mediated activation of the DNA damage response is independent of SLX4, we wanted to confirm that this activation by Vpr still proceeds through ATR in the absence of SLX4 (10). Therefore, we looked in SLX4-deficient cells for G_2_ arrest by Vpr in the presence of the ATR-specific inhibitor VE-821. We found that the level of Vpr-mediated G_2_ arrest was decreased in the presence of the ATR inhibitor in both wild-type and deficient cells (Fig. 5). This indicates that, while SLX4 is not necessary for activation of the DNA damage response by Vpr, ATR is important for this activation for both HIV-1 and HIV-2 Vpr.

### Activation of the DNA damage response is largely conserved.

**FIG 6:**
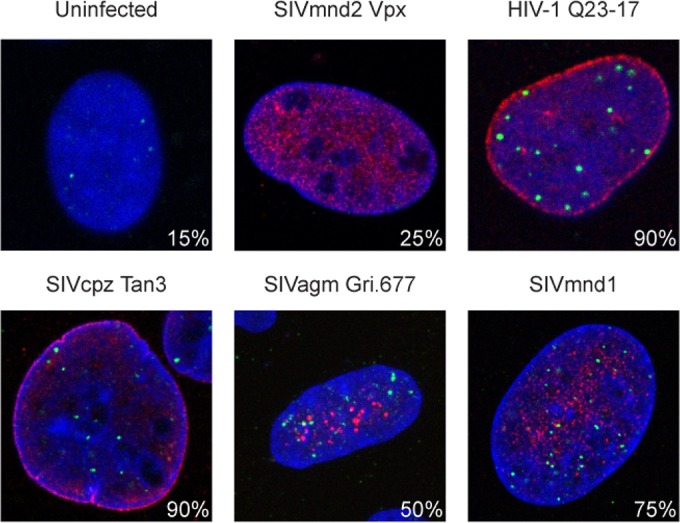
Activation of the DNA damage response is conserved by primate lentiviruses in human cells. Representative immunofluorescence images of U2OS cells expressing 3× FLAG-tagged Vpr or SIVmnd2 Vpx and uninfected controls are shown. FANCD2 foci show activation of the DNA damage response. Blue (DAPI) shows the nuclei, 3× FLAG Vpr is shown in red, and FANCD2 is shown in green. Percentages were calculated as described for Fig. 1A (*n* = >5).

As we found that both HIV-1 and HIV-2 Vpr proteins cause G_2_ arrest and FANCD2 focus formation but that this activation is independent of SLX4, we hypothesized that a conserved function of Vpr is that of activation of the DNA damage response. Therefore, to understand if the ability of Vpr to affect the DNA damage response has been conserved more broadly in the course of primate lentiviral evolution, we assayed for the ability of Vpr orthologs from diverse primate lentiviruses (2) to cause accumulation of FANCD2 foci in human cells. Each Vpr protein was expressed individually via AAV vectors in U2OS cells, and FANCD2 foci were analyzed by indirect immunofluorescence. We showed that, in addition to Vpr from HIV-1 LAI and HIV-2 Rod9 (Fig. 1), HIV-1 Q23-17, SIVcpz Tan3, SIVagm.gri, and SIVmnd1 Vpr proteins also increased FANCD2 focus formation, whereas Vpx from SIVmnd2 did not (Fig. 6). Importantly, neither HIV-1 Q23-17 nor SIVcpz Tan3 can recruit SLX4 (Fig. 2A and C, respectively) and yet both increase FANCD2 focus formation in human cells. While the inability of some Vpr orthologs to activate the DNA damage response may be due to species-specific differences, our data suggest that activation of the DNA damage response is a conserved and important function of lentiviral Vpr.

## DISCUSSION

Here we showed that activation of the DNA damage response is independent of SLX4 recruitment, as Vpr isolates that do not interact with SLX4 still activate the DNA damage response. Further support for the idea of this independence of function was seen in the ability of Vpr to activate the DNA damage response following CRISPR/Cas9 knockout of SLX4. On the other hand, despite the wide divergence of primate lentiviruses, activation of the DNA damage response is a conserved function and is therefore possibly related to an important function of these viruses. Furthermore, our data indicate that how and why Vpr activates the DNA damage response remains unknown.

### SLX4 is not necessary for activation of the DNA damage response by Vpr.

While we found activation of the DNA damage response to be conserved, our results suggest that SLX4 recruitment by Vpr is not conserved by HIV-1, HIV-2, or closely related viruses. This result is distinct from those described in previous reports showing that SLX4 recruitment is conserved by some Old World monkey Vpr orthologs (20). One possible explanation for this difference is that the ability to recruit SLX4 arose early in Vpr evolution and has been maintained by many of the SIV strains infecting Old World monkeys but was lost by the ancestors of both HIV-1 and HIV-2 (SIVrcm and SIVmus strains and SIVsmm strains, respectively). It is also possible that the interaction with SLX4 was gained and lost multiple times during primate lentiviral evolution. However, our data showing that multiple closely related isolates of HIV-1 Vpr are variable in their ability to interact with SLX4, despite relatively high amino acid similarity between these HIV-1 group M Vpr proteins, suggest that this interaction is possibly random and nonspecific.

It is possible that the interaction of Vpr with SLX4 is a result of Vpr recruiting (and degrading) Mus81 (14, 20, 33), an SLX4-binding protein. It was recently shown that SLX4 is not required for the degradation of Mus81 by Vpr (34) and that Mus81 degradation does not correlate with G_2_ cell cycle arrest (33–35). This is consistent with our data showing that recruitment of SLX4 is not required for Vpr-mediated cell cycle arrest. Future studies investigating the ability of additional HIV-1 Vpr isolates and orthologs to degrade Mus81 could help shed light on the possible connections of these interactions and their role in the lentiviral life cycle.

Our data showing that SLX4 knockout does not influence the ability of Vpr to activate the DNA damage response, regardless of the ability of the Vpr protein to recruit SLX4, is also different from previous reports of studies performed using either an small interfering RNA (siRNA) approach to reduce SLX4 levels (20) or patient cells expressing a mutated nonfunctional SLX4 (14). It is possible that the differences observed were due to low levels of SLX4 expression for the siRNA experiments (or the presence of a hypomorphic SLX4), which could result in a different phenotype from that seen in the complete absence of SLX4 in the CRISPR knockout experiments. Alternatively, it is possible that, during the time needed to generate the SLX4 CRISPR/Cas9 knockout, all clones from both cell lines adapted to using a different complex to activate ATR and downstream phenotypes. Despite these differences, the lack of correlation between Vpr isolates and orthologs that do not interact with SLX4 and yet still activate the DNA damage response (such as HIV-2) supports the notion that SLX4 recruitment is not required for activation of the DNA damage response by Vpr. The differences seen between HIV-1 and HIV-2 are also congruent with recent reports indicating that these distinct viruses engage the DNA damage response through unique methods (35).

### Species specificity of Vpr functions.

Most interactions of lentiviral accessory proteins with host factors are characterized by distinct species specificities; due to the evolutionary arms race of viral and host proteins, the viral accessory protein from one species has adapted specifically to the host factor found in the species that it infects. This is most prominent in the arms race between accessory proteins and host restriction factors. For example, Vif from HIV-1 is unable to interact with and degrade APOBEC3G from some other primates due to rapid evolution of APOBEC3G and the adaptation of HIV-1 Vif specifically to hominid APOBEC3G (36, 37). Similar species-specific virus-host interactions occur with Vpx-SAMHD1, CA-Trim5a, and Nef/Vpu-Tetherin (21). Interestingly, the ability of diverse lentiviruses to activate the DNA damage response in human cells indicates that there is a lack of species specificity.

These results are somewhat in contrast to those of two earlier papers which suggested that G_2_ arrest by Vpr was species specific (30, 38). However, both of those earlier studies used only a limited number of Vpr strains; using a broader panel, we found that Vpr from most of the SIVs that we tested does indeed cause DNA damage in a human cell line. This lack of species specificity for Vpr suggests that the host interactions through which Vpr causes DNA damage and cell cycle arrest are likely to be highly conserved between primate species, as is the case for human DCAF1, which is bound by diverse Vpr orthologs. This lack of species specificity also shows similarity to the recently identified interaction of the lentiviral protein Nef and the host restriction factor SERINC5 (39–41).

### The role of DNA damage in the HIV lifecycle.

Taking the data together, our evolutionary approach indicates that the primary function and target of Vpr are still unknown. While activation of the DNA damage response is conserved, whether this is the principal function of Vpr or a consequence of an interaction of Vpr with other unknown host factors remains to be determined. It is also possible that activation of the DNA damage response is an unintended bystander consequence of a yet-to-be-identified, conserved, primary function of Vpr. However, our data support the hypothesis that the primary role of Vpr involves either direct or indirect engagement of the DNA damage response (11, 35). This hypothesis is further supported by the ability of Vpr to interact with and degrade multiple proteins involved in the DNA damage response, including UNG2 (42), HLTF (35, 43), and Mus81 (14, 33), in addition to the interaction with the CRL4^DCAF1^ ubiquitin ligase complex, which has a primary role in DNA damage response pathways (44–47). But it is still unclear how or why lentiviruses have conserved this function. Together, the results from our study and others highlight the conservation and importance of engagement of the DNA damage response by Vpr through a mechanism that is independent of engagement with SLX4.

## MATERIALS AND METHODS

### Plasmids.

pCDNA 3× FLAG Vpr constructs were described previously (2, 22). The following Vpr sequences were synthesized as Gblocks (IDT) and subcloned into the pCDNA 3× FLAG construct using standard cloning techniques: SE6165, SE9280, ETH2220, SL92B, CFU212, MB897, EK505, Cam13, and CP684. The following consensus Vpr sequences were used: HIV-1 N Vpr (MERAPEDAGPQREPYNEWALELLEELKNEAVRHFPRIWLHGLGQHIYNTYGDTWEGVEAIIRILQQLLFIHYRIGCQHSRIGITPQRRRNGASRS) and HIV-1 O Vpr (MEQAPEDQGPAREPFNEWALELLEELKAEAVRHFPRPWLQALGQYIYETYGDTWVGVMAIIRILQQLLFTHFRIGCQHSRIGINPSNTRGRGRRNGSSRS).

For AAV expression of Vpr, GFP-T2A-3× FLAG Vpr sequences were synthesized (IDT) and cloned into pscAAV-CMV-GFP (48) (generously provided by Dan Stone, Fred Hutchinson Cancer Research Center) by Gibson Assembly (NEB). Lenti-CRISPR V2, psPAX2, and pMD2.G were purchased from Addgene. Guide sequences were synthesized (IDT) and cloned into LentiCRISPR V2 following the Zhang laboratory protocol (49). The guide sequences used were 5′-CACCGTCCTCACAGCCGCTGTTGC-3′ and 5′-AAACGCAACAGCGGCTGTGAGGAC-3′.

HA ΔN SLX4 was kindly provided by Wade Harper (Harvard Medical School) (32).

### Transfection.

293T and U2OS cells were transfected with TranIT-LT (Mirus Bio) according to the instructions of the manufacturer. Titrations of Vpr constructs were performed in order to normalize for expression, and 5 µg of ΔN SLX4 cells was transfected in coexpression experiments. The total amount of DNA in all transfections was maintained at a constant level with appropriate empty vectors.

### Immunoprecipitations and Western blotting.

293T cells were transfected by TransIT-LT1 (Mirus Bio) with the appropriate plasmids 36 h prior to lysis. For whole-cell lysates, 293T cells were lysed in radioimmunoprecipitation assay (RIPA) buffer for 10 min on ice and were cleared at 15,000 × *g* for 10 min. For immunoprecipitations, cells were washed twice with phosphate-buffered saline (PBS) and lysed with RIPA buffer plus protease inhibitor cocktail (Roche) for 30 min in an ice-cold sonicator bath. Lysates were cleared at 15,500 × *g* for 15 min, and immunoprecipitations were performed for 1 h at 4 C with EZ-view Red anti-HA affinity gel (Sigma-Aldrich). Following immunoprecipitation, the affinity gel was washed four times with IP lysis buffer and proteins were eluted in 2× Laemmli sample buffer and analyzed by Western blotting. Lysates for detection of FLAG-Vpr were run on 12% SDS-PAGE gels, and lysates for detection of HA-SLX4 lysates were run on 7% SDS-PAGE gels. The following antibodies were used: HA-specific antibody (BAbCO), anti-FLAG M2 antibody (Sigma-Aldrich), anti-tubulin antibody (Sigma-Aldrich), and anti-SLX4 antibody (Abnova). Primary antibodies were detected with a corresponding horseradish peroxidase-conjugated secondary antibody (Santa Cruz Biotech).

### Generation of viruses and single-cell clones.

AAV vectors were generated by transient transfection of HEK 293 cells using polyethyleneimine (PEI) as previously described (50). Levels of DNase-resistant vector genomes were quantified by inverted terminal repeat (ITR)-specific quantitative PCR (qPCR) using a linearized plasmid standard according to the method of Aurnhammer et al. (51). Lentiviruses for CRISPR/Cas9 expression were generated by transient transfection of 293T cells as previously described (49). For single-cell clones, infected cells were placed under conditions of puromycin (Sigma-Aldrich) selection (2.5 µg/ml for U2OS cells and 2 µg/ml for 293T cells) 24 h after infection, and cells were sorted as single cells using a BD Influx cell sorter 72 h later and allowed to expand. Wild-type cells were also infected with empty vector control virus, subjected to single-cell cloning, and selected for puromycin. Cells were maintained under conditions of puromycin selection throughout the process.

### Analysis of CRISPR/Cas9 targeted clones.

Genomic DNA was extracted using QuickExtract DNA extraction solution (Illumina), the targeted loci were amplified by PCR of the genomic DNA using Hurculase II Fusion DNA polymerase (Agilent), and the PCR products were Sanger sequenced. To assess variability of alleles, genomic PCR products were cloned into the pGEM-T Easy vector (Promega) and 10 colonies from each plate were picked and directly Sanger sequenced.

### Indirect immunofluorescence.

Indirect immunofluorescence was performed as previously described (52) with minor modifications. Briefly, cells were plated on cover slides in 24-well plates at 5 × 10^4^ cell/well and allowed to settle overnight. Cells were then infected with AAV2 at equal titers for 24 to 36 h. Following infection, the cells were permeabilized with 0.5% Triton X-100 (Thermo Fisher) for 5 min on ice and then fixed in 4% paraformaldehyde (Santa Cruz) for 20 min. Cells were incubated with the appropriate primary antibody (anti-FLAG M2 antibody [Sigma-Aldrich] or anti-FANCD2 antibody [Abcam]) and Alexa Fluor-conjugated secondary antibodies (Life Technologies). 4′,6-Diamidino-2-phenylindole (DAPI; Life Technologies) was used to stain the nuclei. Microscopy was performed using a Leica TCS-SP5 microscope, and results were analyzed using LAS-X software.

### Cell cycle and cell survival analysis.

293T or U2OS cells were plated in a 6-well dish at 3 × 10^5^ cells/well and infected with equal titers of AAV2 for 20 h. Cells were fixed with ice-cold ethanol and permeabilized with 0.2% Tween 20–PBS–0.1% bovine serum albumin (Sigma-Aldrich). DNA was stained with 0.01 g/ml propidium iodide (Sigma-Aldrich)–RNase A–PBS. Cell cycle status, as measured by DNA content, was assessed by flow cytometry on a FACSCanto II system (BD) and analyzed with FlowJo software. Cell survival was measured by a crystal violet absorbance-based assay as previously described (52).

## SUPPLEMENTAL MATERIAL

Figure S1  HIV-1 and HIV-2 Vpr both cause G_2_ cell cycle arrest. U2OS cells were infected with AAV vectors containing HIV-1 Vpr, HIV-2 Vpr, or no Vpr or were left uninfected and were assayed for cell cycle status by flow cytometry. Data are related to Fig. 1. Download Figure S1, PDF file, 0.5 MB

Figure S2  Diverse Vpr orthologs show variability in their recruitment of SLX4. (A and B) Schematic phylogenetic representation of Vpr orthologs used in this study. The tree is not drawn to scale. Phylogeny data are based on results published by Sharp and Hahn (24) as well as on fast statistical alignment (FSA) of Vpr sequences. (C, D, and E) Dark exposures of FLAG-Vpr from the HA-SLX4 immunoprecipitation whose results are presented in Fig. 2. Download Figure S2, PDF file, 0.2 MB

Figure S3  CRISPR/Cas9 knockout of SLX4 in 293T cells does not alter the ability of HIV-1 or HIV-2 Vpr to cause cell cycle arrest. (A) Results of sequencing of genomic DNA from wild-type 293T cells (WT) as well as two single-cell clones (SLX4 KO1 and SLX4 KO2) are shown. The Cas9 guide region is boxed. (B) 293T WT, KO1, and KO2 cells were infected with AAV vectors containing HIV-1 or HIV-2 Vpr and assayed for cell cycle status. Virus without Vpr was used as a negative control. Data are related to Fig. 3 and 4. Download Figure S3, PDF file, 0.3 MB
