# Supplementary figures and images for: Activation of the DNA Damage Response Is a Conserved Function of HIV-1 and HIV-2 Vpr That Is Independent of SLX4 Recruitment
Source: mBio. 2016 Sep 13;7(5):e01433-16. doi: 10.1128/mBio.01433-16 (PMC5021806; doi:10.1128/mBio.01433-16)

Figure S1

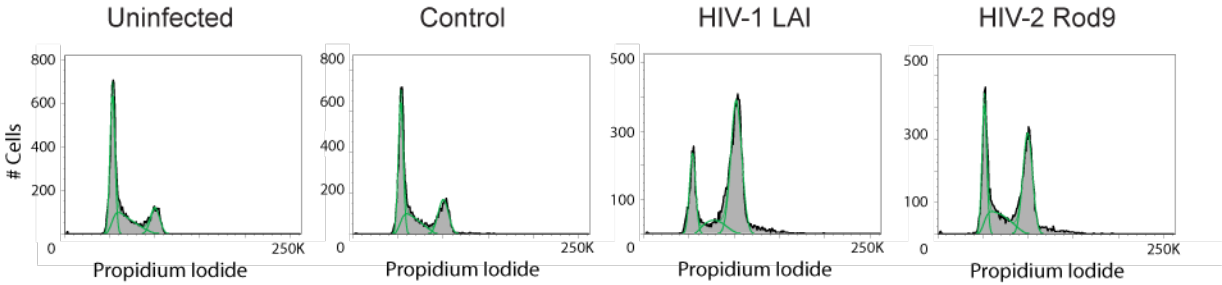

Supplement: Figure S1 — HIV-1 and HIV-2 Vpr both cause G2 cell cycle arrest. U2OS cells were infected with AAV vectors containing HIV-1 Vpr, HIV-2 Vpr, or no Vpr or were left uninfected and were assayed for cell cycle status by flow cytometry. Data are related to Fig. 1. Download [file mbo004162989sf1.pdf]

Figure S2

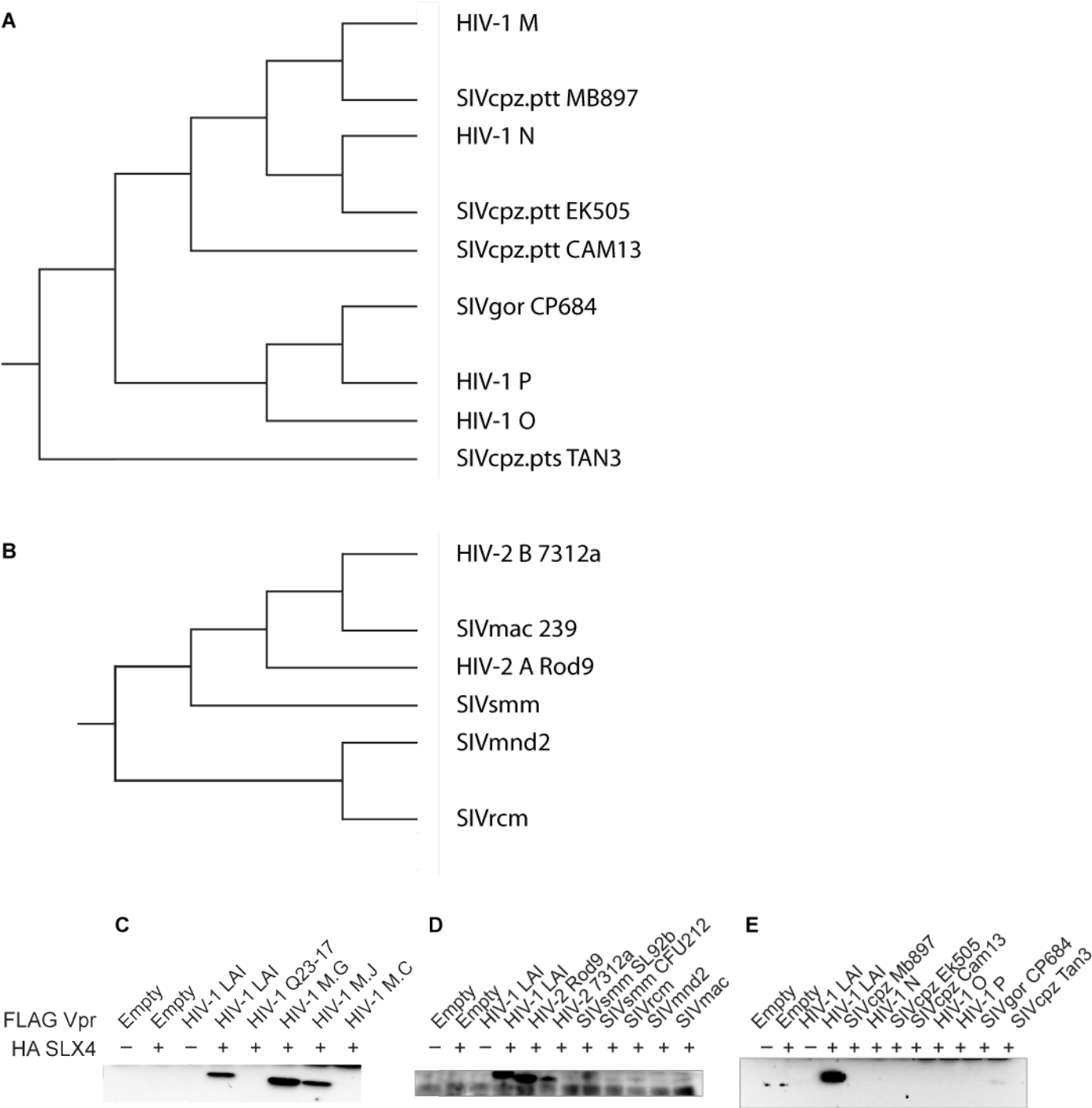

Supplement: Figure S2 — Diverse Vpr orthologs show variability in their recruitment of SLX4. (A and B) Schematic phylogenetic representation of Vpr orthologs used in this study. The tree is not drawn to scale. Phylogeny data are based on results published by Sharp and Hahn (24) as well as on fast statistical alignment (FSA) of Vpr sequences. (C, D, and E) Dark exposures of FLAG-Vpr from the HA-SLX4 immunoprecipitation whose results are presented in Fig. 2. Download [file mbo004162989sf2.pdf]

Figure S3

A

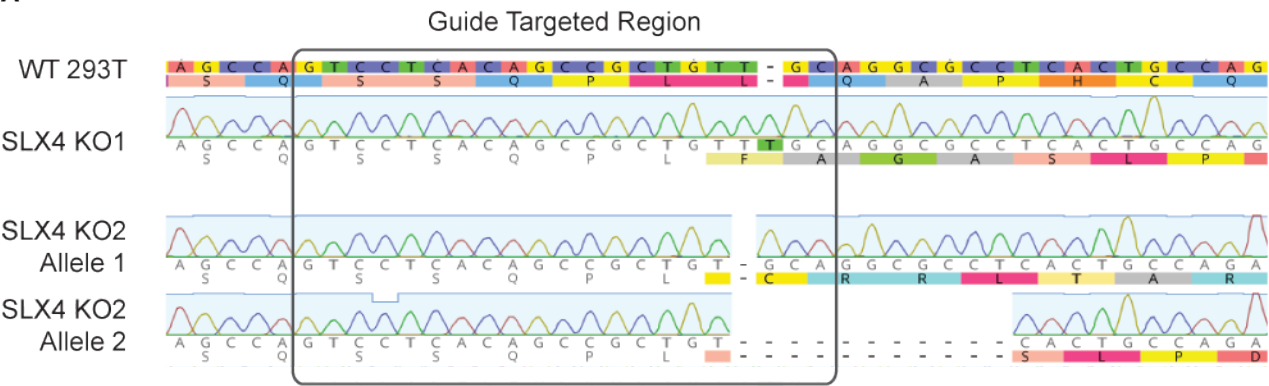

B

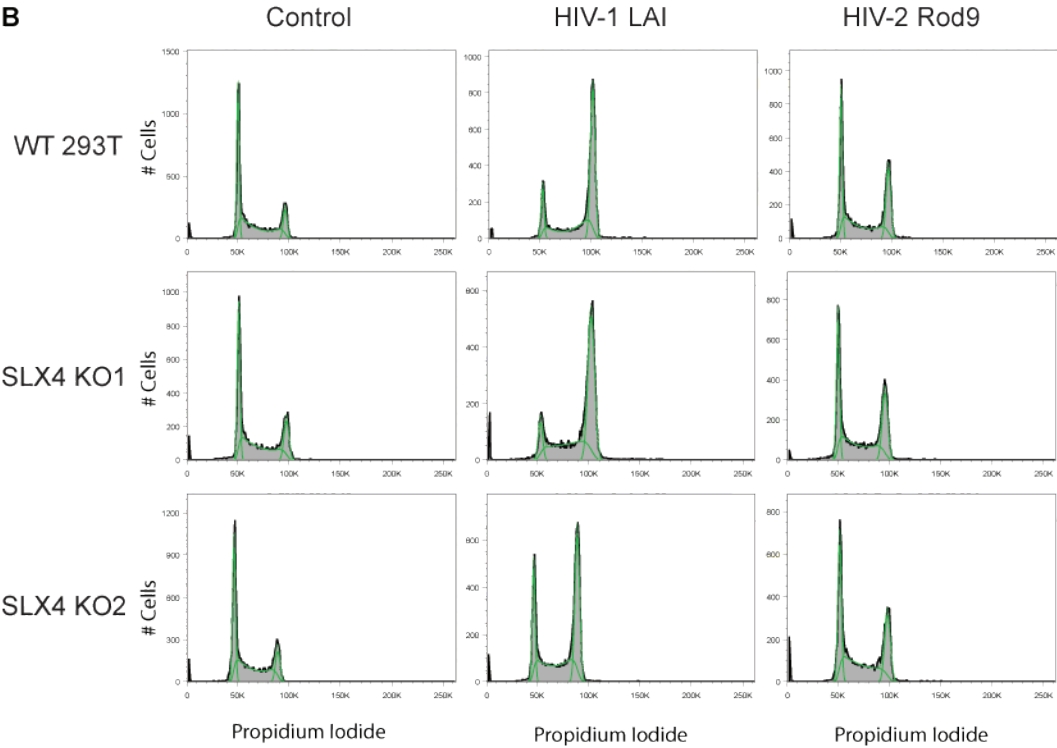

Supplement: Figure S3 — CRISPR/Cas9 knockout of SLX4 in 293T cells does not alter the ability of HIV-1 or HIV-2 Vpr to cause cell cycle arrest. (A) Results of sequencing of genomic DNA from wild-type 293T cells (WT) as well as two single-cell clones (SLX4 KO1 and SLX4 KO2) are shown. The Cas9 guide region is boxed. (B) 293T WT, KO1, and KO2 cells were infected with AAV vectors containing HIV-1 or HIV-2 Vpr and assayed for cell cycle status. Virus without Vpr was used as a negative control. Data are related to Fig. 3 and 4. Download [file mbo004162989sf3.pdf]
